# Machine learning and mechanistic studies on *p*-nitrophenol remediation using sustainable activated carbon

**DOI:** 10.1038/s41598-026-42718-2

**Published:** 2026-03-04

**Authors:** Ashalakshmi Kodandoor, Gokulakrishnan Murugesan, Thivaharan Varadavenkatesan, Ramesh Vinayagam, Raja Selvaraj

**Affiliations:** 1 Manipal Institute of Technology, Manipal Academy of Higher Education, Manipal, India; 2 Department of Biotechnology, M.S. Ramaiah Institute of Technology, Bengaluru, 560054 Karnataka India

**Keywords:** Activated carbon, Machine learning, *Pistacia vera*, *p*-nitrophenol, Wastewater treatment, Chemistry, Engineering, Environmental sciences, Materials science

## Abstract

This study presents a sustainable approach for *p*-nitrophenol (*p*NP) removal by synthesizing activated carbon with a large surface area from waste *Pistacia vera* shells using H_3_PO_4_ activation. Comprehensive characterization confirmed a high specific surface area (670.25 m^2^ g^− 1^) and a heterogeneous structure rich in functional groups, which are beneficial for adsorption. Adsorption followed pseudo-second-order kinetics, and the equilibrium dataset followed the Langmuir isotherm, with a maximum adsorption capacity of 142.93 mg g^− 1^. The thermodynamic results showed that the adsorption was a spontaneous and exothermic (− 13.43 kJ mol^− 1^) process. ANN and ANFIS models were developed to predict the adsorption behavior. The ANFIS model exhibited the best predictive performance, with an R^2^ of 0.9935. ANFIS sensitivity analysis identified that contact time and initial *p*NP concentration were the key factors. Regeneration tests demonstrated that the adsorbent could be reused for five cycles, supporting its practical applicability. Real water matrix studies have demonstrated robust *p*NP removal in real-world water scenarios, highlighting its environmental relevance. Thus, waste-derived adsorbents present a low-cost and sustainable option for *p*NP removal.

**Supplementary Information:**

The online version contains supplementary material available at 10.1038/s41598-026-42718-2.

## Introduction

Environmental pollution continues to pose serious risks to living organisms. *p-*Nitrophenol (*p*NP, 4‐nitrophenol, 4‐NP) is a hazardous phenolic compound commonly used in pesticide synthesis, dyes, explosives, pharmaceuticals, and petrochemical products^1^. The United States Environmental Protection Agency has classified *p*NP as a priority pollutant^2^. It usually enters the environment through industrial discharges, laboratory waste, agricultural runoff, and leaching of polluted soils^3^. Water pollution caused by *p*NP has become a serious environmental and health concern because of its wide industrial use and its tendency to persist in water. Once it enters aquatic systems, it stays for long periods since it does not break down easily and remains stable under both acidic and alkaline conditions^4^. Therefore, removing *p*NP from wastewater is important for limiting its toxic effects and protecting aquatic ecosystems. Finding efficient and sustainable treatment methods remains a key challenge in addressing persistent pollutants.

Methods used for removing *p*NP include advanced oxidation processes (AOPs)^5^, biodegradation^6^, photocatalysis^7^, and membrane filtration^8^. Even though many of these techniques show high removal in controlled setups, their use in real practice is often limited. AOPs such as Fenton, ozonation, and electrochemical oxidation usually need expensive reagents^9^, are sensitive to scavenging species, and are still difficult to scale up^10^. Photocatalysis can break down contaminants under light, but it tends to perform poorly in actual wastewater because catalysts lose efficiency and may produce harmful byproducts^11^. Biodegradation is eco-friendly, yet it depends heavily on maintaining strict biological conditions, making it slower and harder to control. Membrane filtration suffers from fouling, flux decline, and increased operating costs due to frequent cleaning or replacement^12^.

Adsorption is a widely recognized method for wastewater treatment. A broad range of adsorbents have been investigated, including activated carbon (AC), biochar, lignocellulosic materials and their derivatives, synthetic nanostructures and polymers, metal-organic frameworks (MOFs), and fibrous peat^13^. Nevertheless, MOFs and their derivatives tend to agglomerate during pyrolysis, which reduces the surface area. Consequently, fewer active sites are available, leading to a decline in overall efficiency. Their weak metal-ligand coordination renders them hydrolytically unstable in aqueous, acidic, or alkaline media^14^. Similarly, certain synthetic nanostructures and polymers also require complex, high-cost syntheses that hinder scale-up^15^. These limitations underscore the need for robust, low-cost, and efficient alternatives, such as biomass-derived AC.

AC has been extensively applied in wastewater treatment and industrial operations because of its cost-effectiveness, ease of production, regenerability, high porosity, and high specific surface area (SSA), which enhance its adsorption performance^16^. The growing interest in converting lignocellulosic biomass into activated carbon reflects a shift toward valorizing waste into sustainable, high-value adsorbents with high efficacy^17^. In this context, AC produced from agricultural residues has been widely investigated for the removal of *p*NP, and nutshells, in particular, have emerged as attractive low-cost precursors^18^. *Pistacia vera* (pistachio) shells are an abundant lignocellulosic agro-waste that can be converted to high-surface-area, well-textured micro-or mesoporous carbons using standard chemical and physical activation routes^19^. Chemical activation to convert lignocellulosic biomass into AC offers improved energy efficiency and operates at lower carbonization temperatures and shorter durations, making it a more effective production method. H_3_PO_4_ is the most commonly used chemical activating agent for creating AC from lignocellulosic biomass. It offers effective recovery, minimal environmental impact, and increased carbon yield^20^. AC derived from cherry stones^21^, date stones^21^, and pili nut shells^22^ have been reported earlier, but these methods often require harsher conditions and high amounts of chemicals.

Machine learning (ML) methods like artificial neural network (ANN) and adaptive neuro-fuzzy inference system (ANFIS) are now commonly employed for adsorption studies because they can map the complex behavior of operating variables and their effect on removal efficiency^23^. ANN learns patterns from experimental data, allowing it to predict adsorption outcomes in systems with many interacting factors. ANFIS combines neural network learning and fuzzy logic, which is useful when the data involve uncertainty or imprecise measurements. Sensitivity analysis carried out through ANFIS has also helped identify which parameters influence the process the most, providing useful guidance for understanding the mechanism and improving optimization strategies.

This study reports the adsorption performance of activated carbon derived from *P. vera* shells (PSAC) using batch tests. Kinetics, equilibrium isotherms, thermodynamics, desorption, and spiking tests were performed to better understand the mechanisms of *p*NP removal and the reusability of the adsorbent. Machine learning models, such as ANN and ANFIS, are also used to predict the adsorption behavior, supporting the experimental results and improving the accuracy of the performance evaluation.

## Materials and methodologies

### Materials

*P. vera* nuts were obtained from a local grocery store. *para*-nitrophenol (C_6_H_5_NO_3_, MW: 139.11, ˃ 98%) was purchased from Himedia, India. H_3_PO_4_ (85%), NaHCO_3_ (99.7%), HCl (35%), and NaOH (97%) were acquired from Loba Chemie, India. Distilled water was used in all the experiments.

### Adsorbent synthesis

The collected *P. vera* nut shells were thoroughly washed with water, rinsed with distilled water, and dried at 80 °C for 12 h. Subsequently, the biomass was crushed into a coarse powder using a mixer grinder. This powder was then mixed with H_3_PO_4_ (1:0.5, w v^− 1^) and kept at room temperature for 6 h. The aged sample was oven-dried at 80 °C, 12 h. The dried substance was subsequently calcined at 400 °C, 2 h in a muffle furnace. Following this, it was washed using distilled water and 1% NaHCO_3_ until neutral pH. Finally, the washed material was dried at 80 °C, 12 h. The resulting AC was designated as PSAC, and the preparation steps are depicted in **Fig. ****S1****.**

### Physicochemical and structural properties of PSAC

Field Emission Scanning Electron Microscopy (FE-SEM, Apreo 2 S Lo-Vac, Thermo Fisher Scientific, USA) was used to examine the surface morphology. Energy‐Dispersive X‐ray Spectroscopy (EDS, Oxford Instruments, UK) was employed to examine the elemental nature. Brunauer‐Emmett‐Teller analysis (BET, Smart Instruments, India) was used to evaluate the SSA and pore volume. Fourier Transform Infrared Spectroscopy (FT‐IR, Shimadzu 8400 S spectrophotometer, Japan) was used to determine the functional groups. X‐ray Diffraction (XRD, D8 Advance diffractometer, Bruker, Germany) analysed the crystalline phases. X-ray Photoelectron Spectroscopy (XPS, Thermo Fisher Scientific, UK) was used to investigate the surface and bonding nature. The point of zero charge (pH_PZC_) of PSAC was determined by the pH drift method.

### Batch adsorption experiment

The pH effect was evaluated by mixing 0.5 g L^− 1^ of PSAC with 100 mL of 50 mg L^− 1^ *p*NP solution, with pH adjusted in the range of 2 to 12. The dosage influence was studied by taking varying dosages of PSAC ranging from 0.1 to 0.5 g L^− 1^ in 25 mg L^− 1^ *p*NP at optimized pH. The influence of the initial concentration of *p*NP was determined by taking different concentrations of *p*NP solution ranging between 10 and 50 mg L^− 1^ with optimized pH and dosage. The temperature effect was evaluated in the range of 20 to 50 °C under optimized pH, dosage, and concentration.

All adsorption tests were done in an orbital shaker set at 30 °C, with continuous agitation maintained at 150 rpm. Post-centrifugation, analysis of the residual *p*NP in the supernatant was done at 317 nm with a UV-Visible spectrophotometer (Shimadzu UV-1900i). Triplicate experiments were done and the mean was considered for analysis. The removal efficiency (R, %) and adsorption capacity (qₑ, mg g^− 1^) were determined using Eqs. (1) and (2).

1 $$R=\frac{{C}_{i}-{C}_{f}}{{C}_{i}}\times100$$

2 $${q}_{e}=\frac{\left({C}_{i}-{C}_{e}\right)V}{m}$$

Where C_i,_ C_f_ and C_e_ correspond to the respective starting, final and equilibrium concentrations of *p*NP (mg L^− 1^). V denotes the solution volume (L), and m represents the PSAC quantity (g).

### Adsorption isotherm, kinetic, and thermodynamic studies

#### Adsorption isotherm modelling

The adsorption behaviour and PSAC performance were evaluated by fitting the data to various isotherm model equations, namely Langmuir, Freundlich, and Temkin, as represented by Eqs. (3), (4), and (5) respectively

3 $${\mathrm{L}\mathrm{a}\mathrm{n}\mathrm{g}\mathrm{m}\mathrm{u}\mathrm{i}\mathrm{r}:q}_{e}=\frac{{q}_{m}{K}_{L}{C}_{e}}{(1+{K}_{L}{C}_{e})}$$

4 $${\mathrm{F}\mathrm{r}\mathrm{e}\mathrm{u}\mathrm{n}\mathrm{d}\mathrm{l}\mathrm{i}\mathrm{c}\mathrm{h}:q}_{e}={K}_{F}{C}_{e}^{\frac{1}{n}}$$

5 $${\mathrm{T}\mathrm{e}\mathrm{m}\mathrm{k}\mathrm{i}\mathrm{n}:q}_{e}={B}_{T}\mathrm{l}\mathrm{n}\left({K}_{t}{C}_{e}\right)$$

Where, K_L_ is the Langmuir constant (L mg^− 1^), and q_m_ is the maximum adsorption capacity (mg g^− 1^). 1/n is the Freundlich exponent, K_F_ is the Freundlich constant ((mg g^− 1^)/(L mg^− 1^)^1/n^), B_T_ refers to the Temkin constant associated with the heat of adsorption (J mol^− 1^), and K_t_ denotes the Temkin equilibrium binding constant (L mg^− 1^). The optimal model was selected based on the higher R^2^ with lower values of reduced chi-square (χ^2^)^24^.

#### Adsorption kinetic studies

Kinetic experiments were conducted to identify the rate-controlling mechanism. The adsorption characteristics were examined by comparing the experimental results with the pseudo-first order (PFO), pseudo-second order (PSO), and intraparticle diffusion (IPD) kinetic models, as given in Eqs. (6)-(8).

6 $$\mathrm{P}\mathrm{F}\mathrm{O}:{q}_{t}={q}_{e}(1-{e}^{-{K}_{1}t})$$

7 $${\mathrm{P}\mathrm{S}\mathrm{O}:q}_{t}=\frac{{q}_{e}^{2}{K}_{2}t}{1+{q}_{e}{k}_{2}t}$$

8 $${\mathrm{I}\mathrm{P}\mathrm{D}:q}_{t}={K}_{diff}{t}^{0.5}+C$$

The parameters qₜ and qₑ stand for the adsorption capacities at time t and at equilibrium, respectively. K_1_ represents the PFO rate constant (min^− 1^), K_2_ denotes the PSO rate constant (g mg^− 1^ min^− 1^), and K_diff_ represents the rate constant of IPD ((mg g^− 1^) min^0.5^), while C indicates the intercept in the IPD model (mg g^− 1^).

#### Adsorption thermodynamics

To examine the practicability, spontaneity, and nature of the process of adsorption, thermodynamic parameters, which include standard enthalpy change (ΔH°), standard entropy change (ΔS°), and standard Gibbs free energy change (ΔG°) were determined with the Van’t Hoff equation, as shown in Eq. (9).

9 $${K}_{T}=\mathrm{e}\mathrm{x}\mathrm{p}\left[\left(\frac{{\Delta}S^\circ}{R}\right)-\left(\frac{\varDelta H^\circ}{R}\right)\frac{1}{T}\right]$$

Where K_T_ is obtained experimentally as q_e_/C_e_ (L g^− 1^), represents the equilibrium constant, R is the ideal gas constant, and T denotes the absolute temperature (K), and ΔG° is expressed as − RT ln K_T_.

### Reusability studies

Desorption studies were done to examine the reusability of PSAC, with the goal of improving its economic feasibility. Specifically, for regeneration, the used PSAC from adsorption runs (25 mg L^− 1^ *p*NP, 0.2 g L^− 1^ PSAC) was added to 50 mL of 0.1 N NaOH for treatment as the eluent. After desorption, the PSAC was recovered and rinsed repeatedly with distilled water till neutral pH and dried at 80 °C for 12 h. Five sequential adsorption–desorption runs were carried out to examine the reusability of PSAC, and the regenerated adsorbent’s efficiency was evaluated after every cycle.

### Performance of PSAC to remove *p*NP from various water matrices

Water samples were collected from four different sources, namely, tap water (TW), Manipal Lake (ML), well water (WW), and Suvarna River (SR), by the grab sampling method. All samples were spiked with 25 mg L^− 1^ of *p*NP to assess the performance of the adsorbent under natural environmental conditions. The inclusion of various water matrices provides a practical evaluation of the efficiency of PSAC in complex and potentially contaminated environments that are relevant to industrial applications. To ensure accuracy and reproducibility, batch adsorption experiments were done in triplicate for each water type. 0.2 g L^− 1^ PSAC was mixed with 100 mL of the spiked sample. Furthermore, the pH of all samples was uniformly adjusted to 6 and maintained at 30 °C. The adsorption potential of PSAC was then evaluated following the procedures described in the earlier sections.

### Machine learning modelling

#### ANN modelling

**Fig. 1:**
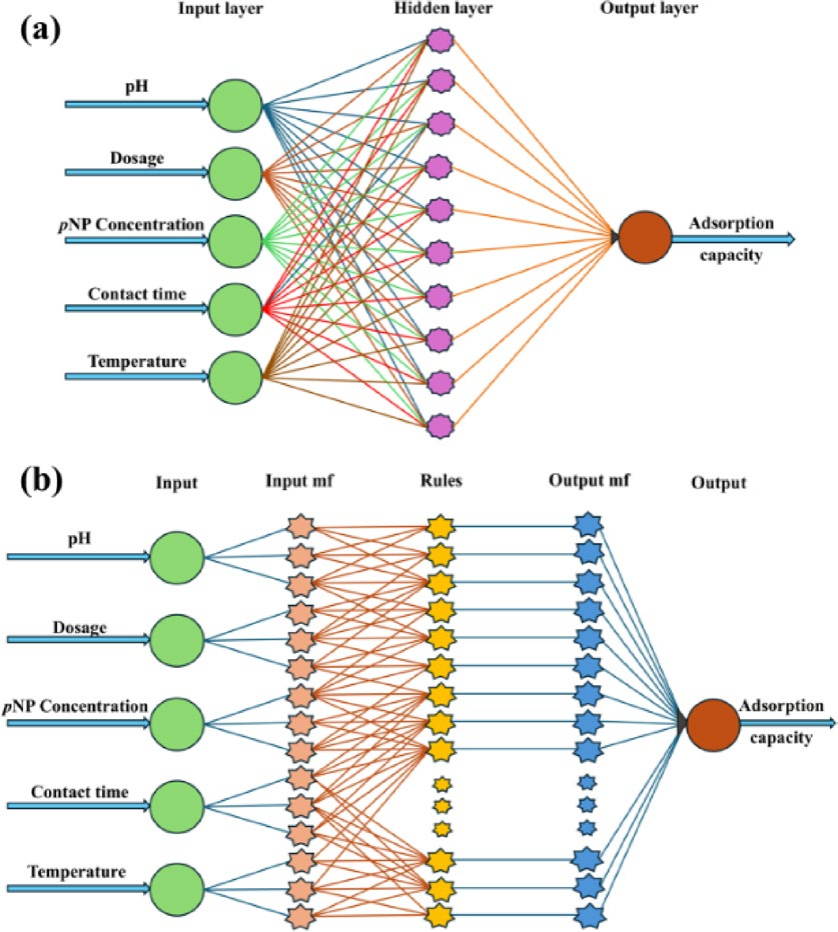
**(a)** ANN and **(b)** ANFIS models for predicting adsorption capacity using process parameters.

An ANN model was developed in MATLAB R2020b (The MathWorks Inc.) to predict the q_e_ of *p*NP onto PSAC. The model followed a three-layer feed‐forward architecture with five input neurons (pH, dosage, initial concentration, contact time, and temperature), a hidden layer optimized through trials with 2‐12 neurons, and one output neuron representing q_e_ (Fig. 1a). The hidden layer employed a tangent sigmoid (tansig) activation function, whereas a linear (purelin) function was used in the output layer. Network training was carried out using the Levenberg‐Marquardt backpropagation algorithm. The dataset of 180 experimental points was randomly divided into 70% (training), 15% (testing), and 15% (validation).

#### ANFIS model

An ANFIS model was developed in MATLAB R2020b (The MathWorks Inc.) to predict the q_e_ of *p*NP onto PSAC. The model combines neural network learning with fuzzy logic reasoning, where the first and last layers denote the inputs and output, respectively, and the intermediate layers correspond to a first-order Sugeno inference system^24^. The structural framework of the ANFIS model is illustrated in Fig. 1b. The input variables were transformed into membership values to generate fuzzy rules, which were then mapped to the output representing q_e_. Training was carried out using a hybrid learning algorithm integrating backpropagation with least-squares estimation, with a maximum of 10 learning epochs. The same ANN dataset was used, with 70% for training and 15% each for testing and validation. The sensitivity analysis was performed to assess the relative impact of the input parameters on q_e_.

#### Comparative evaluation of ANFIS and ANN models

The selection of the most suitable machine learning model was carried out by evaluating statistical performance indicators, as defined in Eqs. (10)-(13).

10 $${R}^{2}=1-\frac{\sum_{i=1}^{n}{\left[{q}_{e,experimental}-{q}_{e,predicted}\right]}^{2}}{\sum_{i=1}^{n}{\left[{q}_{e,experimental}-{q}_{e,average}\right]}^{2}}$$

11 $$MSE=\frac{1}{n}\sum_{i=1}^{n}{\left[{q}_{e,experimental}-{q}_{e,predicted}\right]}^{2}$$

12 $$RMSE=\sqrt{\frac{1}{n}\sum_{i=1}^{n}{\left[{q}_{e,experimental}-{q}_{e,predicted}\right]}^{2}}$$

13 $$MAE=\frac{1}{n}\sum_{i=1}^{n}\left|{q}_{e,experimental}-{q}_{e,predicted}\right|$$

Where n represents the number of data points used in the analysis. The optimal model was identified based on the low values of MSE, RMSE, and MAE, as well as the high R^2^ value, ensuring accurate predictions and reliable data interpretation.

## Results and discussions

### Characterisation of PSAC

#### Morphological features and elemental analysis of PSAC

**Fig. 2:**
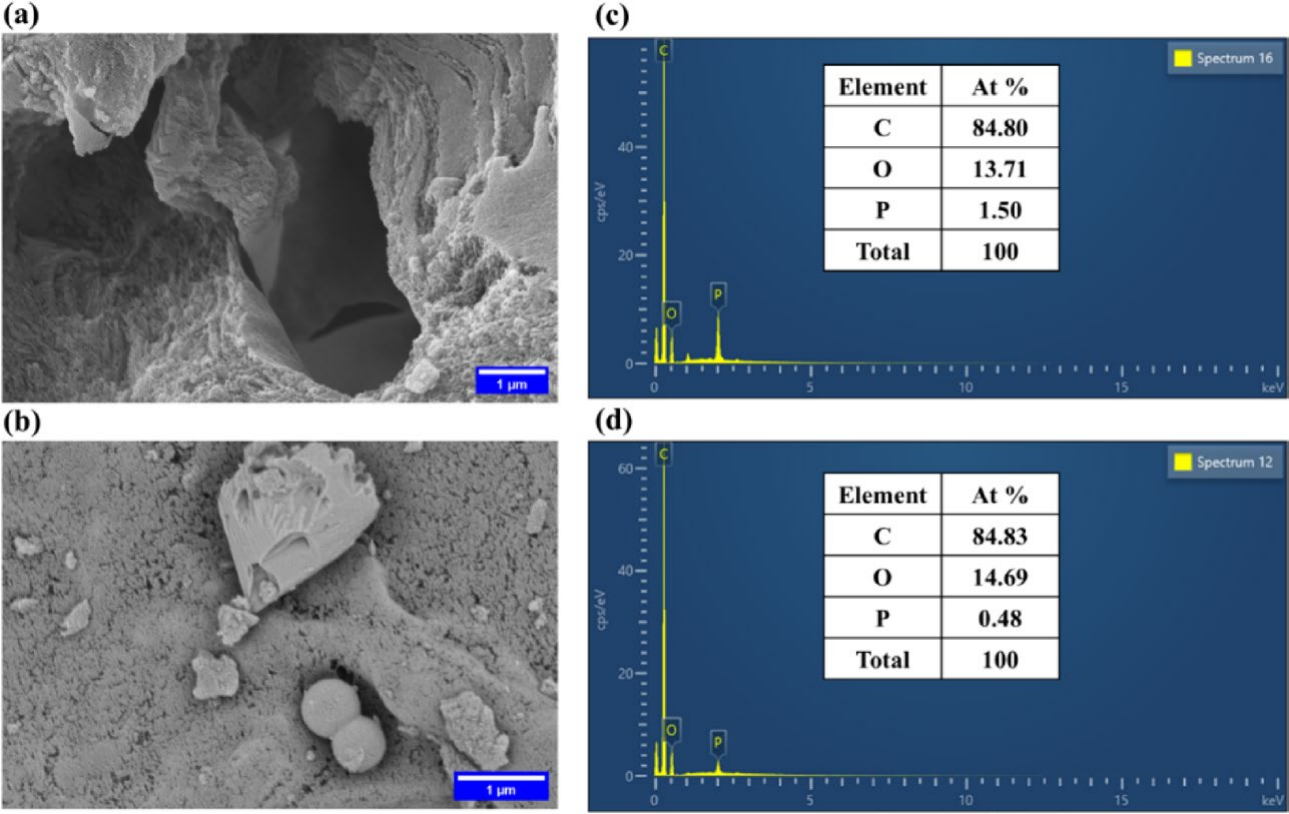
Surface morphology of PSAC observed via FE-SEM **(a)** before adsorption **(b)** after adsorption. EDS spectrum showing the elemental composition of PSAC **(c)** before adsorption **(d)** after adsorption.

FE-SEM was used to study the structural features of PSAC before and after adsorption (Fig. 2**)**. In the pristine sample (Fig. 2a), a highly porous structure with numerous interconnected voids and a rough surface texture was observed. This type of well-developed porosity is crucial for effective adsorption, as it provides abundant sites where adsorption can take place^22^. Post-adsorption (Fig. 2b**)** shows a visibly smoother surface, and many pores appear to be either blocked or covered. The observable difference from a highly porous, rough texture to a smoother, pore-covered surface is consistent with findings reported for other adsorbents, such as activated carbon beads^25^ and graphene mesosponge^26^, further supporting the FE-SEM evidence for adsorbate uptake.

EDS facilitated a precise elemental analysis, enabling the identification and elemental distribution within the samples. In the pristine sample (Fig. 2c), carbon is 84.80%, oxygen 13.71%, and phosphorus 1.50%. After adsorption (Fig. 2d), the oxygen content increased to 14.69%, whereas the phosphorus content decreased to 0.48%. The observed rise in oxygen concentration after adsorption is likely due to the presence of oxygen-containing moieties from the *p*NP molecules interacting with the adsorbent surface.

#### Brunauer-emmett-teller analysis

PSAC showed a high SSA of 670.25 m^2^ g^− 1^ with 0.9853 cc g^− 1^ pore volume and an average pore size of 5.88 nm. Such pores are beneficial for *p*NP adsorption because they provide enough space for *p*NP molecules to diffuse and attach to the internal surface. The presence of mesopores also improves access to the active sites, leading to more efficient adsorption. The SSA value is notably high, particularly when compared with recent findings on AC obtained from cherry (450.4 m^2^ g^− 1^) and date stones (350 m^2^ g^− 1^)^21^ and waste orange peels (540.61 m^2^ g^− 1^)^27^.

#### FT-IR analysis

Key changes before and after adsorption were identified through FT-IR spectral analysis (**Fig. S2a**), demonstrating notable shifts, which indicates the key role of functional moieties in adsorption. The O-H stretching signal shifted from 3294 to 3404 cm^− 1^, falling within the characteristic range of 3200–3550 cm^− 1,^ typical of aliphatic hydroxyl stretching vibrations^28^. A shift in the C-H region from 2913 to 2892 cm^− 1^ is linked to the stretching of aliphatic -CH bonds, suggesting subtle changes in the hydrocarbon environment of the adsorbent surface^29^. The signals observed at 2084 and 2136 cm^− 1^ correspond to alkyne (C ≡ C) stretching vibrations, and the shift in this region after adsorption suggests that these surface functional groups participated in the interaction with *p*NP^30^. The aromatic C = C signal shifted from 1476 to 1512 cm^− 1^ after adsorption, falling within the 1600–1400 cm^− 1^ range associated with the vibrational stretching of aromatic C = C bonds and asymmetric bending of CH_2_ or CH_3_ groups, indicating π‐π stacking between the carbon surface and the aromatic moiety of *p*NP^31^. The band related to the C = O stretching of ketones or carboxylates, which shifted from 1532 to 1610 cm^− 1^, confirms the participation of carbonyl-containing groups during adsorption, likely through hydrogen bonding or electron-donor–acceptor mechanisms^32^. The C-O stretching vibration shifted from 1095 to 1157 cm^− 1^, indicating the contribution of phenolic groups and confirming the interactions among the oxygenated functionalities of *p*NP with the PSAC surface^33^.

#### XRD analysis

XRD patterns of PSAC before and after *p*NP adsorption (**Fig. S2b**) display a broad peak around 2θ = 24°, corresponding to the (002) plane. This reflects the presence of graphite-like microcrystallites within an amorphous matrix^34^. The broad nature of the peak confirms that the material remains largely amorphous even after adsorption. The change in the intensity of the diffraction peak indicated that the PSAC structure was disorganized, possibly due to the accumulation of *p*NP on the PSAC surface^35^.

#### XPS analysis

**Fig. 3:**
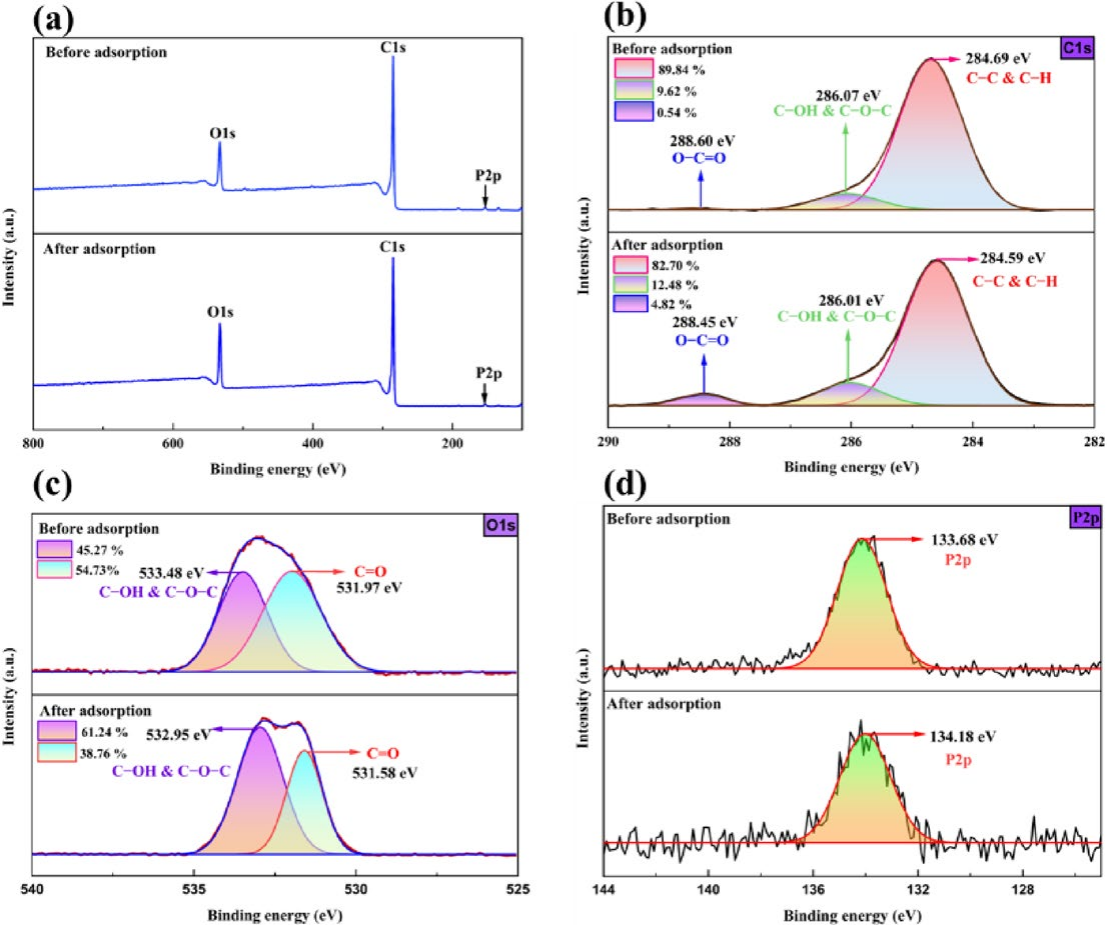
XPS profiles of PSAC before and after *p*NP adsorption: **(a)** survey spectrum, **(b)** deconvoluted C1s, **(c)** deconvoluted O1s, and **(d)** deconvoluted P2p spectra.

The XPS spectrum of PSAC (Fig. 3a) reveals notable peaks associated with carbon, oxygen, and phosphorus (C1s, O1s, P2p), confirming the presence of these elements at the surface. The strong C1s signal confirms the dominance of carbon in PSAC, and the O1s signal indicates the existence of oxygenated functional moieties formed through thermal activation. The P2p peak is associated with phosphatic phosphorus, attributed to the use of H_3_PO_4_ in the adsorbent preparation^36^. The high-resolution C1s spectra show peaks corresponding to C-C & C‐H (284.69 eV), C‐OH & C‐O‐C (286.07 eV), and O‐C = O (288.60 eV) (Fig. 3b). Shifts in the peak intensities and binding energies following adsorption indicate interactions with the adsorbate^37^. The O1s deconvolution (Fig. 3c) reveals peaks attributed to C‐OH, C‐O‐C (533.48 eV), and C = O (531.97 eV). After adsorption, the shift in the binding energies suggests clear interactions between the surface and adsorbed species^38^. The P2p spectra also show a P2p peak moving from 133.68 eV to 134.18 eV after adsorption (Fig. 3d), indicating that the remaining phosphate on the PSAC surface participates in the adsorption process^39^. These spectral changes collectively confirm successful adsorption and surface modification.

### Adsorption results

#### pH effect

**Fig. 4:**
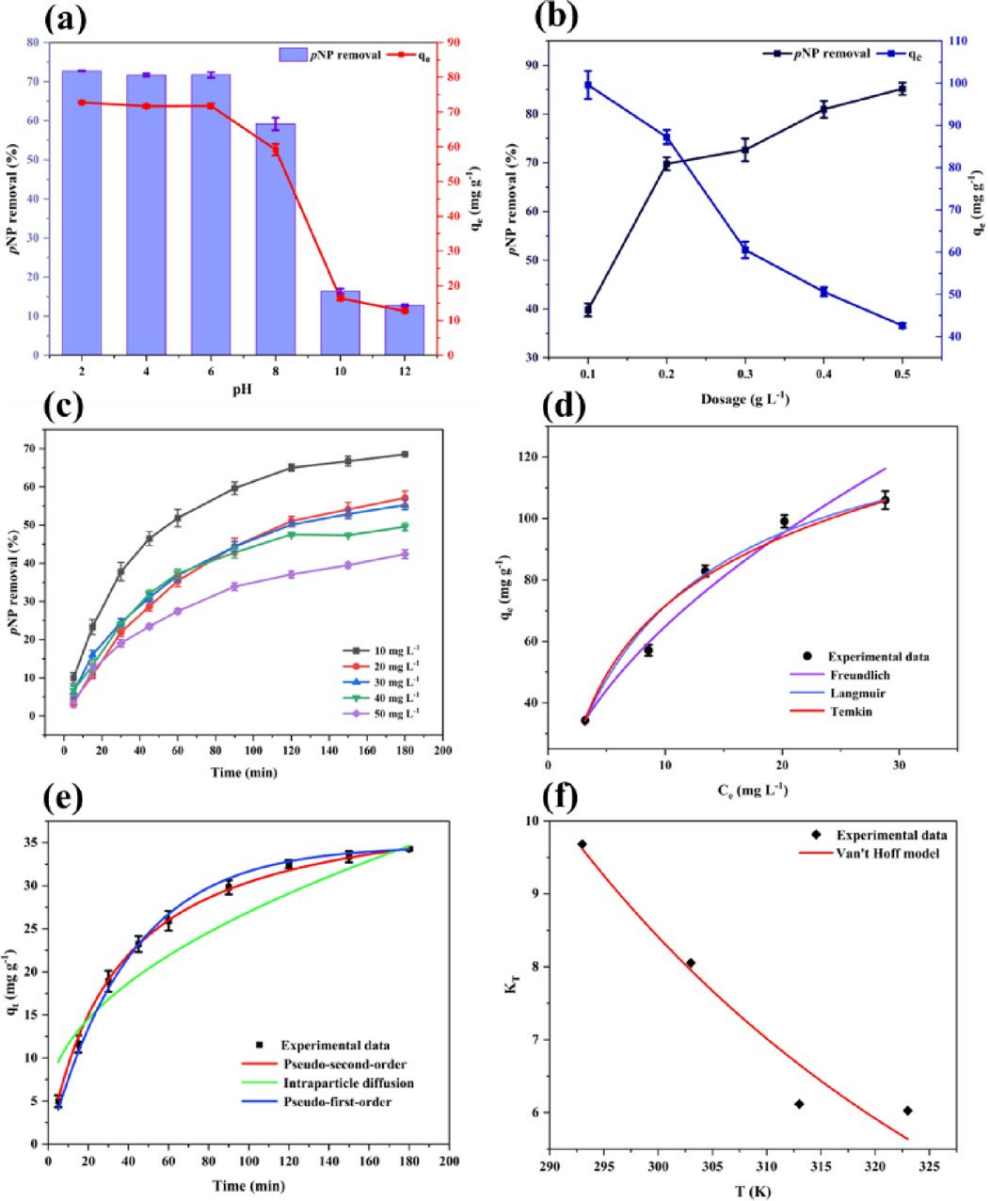
Effect of key parameters on *p*NP adsorption onto PSAC: **(a)** Effect of adsorbate pH, **(b)** adsorbent dosage, **(c)** initial *p*NP concentration, **(d)** Isotherm models for *p*NP adsorption onto PSAC, **(e)** kinetic models describing the adsorption process, and **(f)** Van’t Hoff plot illustrating the thermodynamic behaviour of adsorption.

Figure 4a depicts the pH effect on *p*NP adsorption, where both the %R and q_e_ reached high values under acidic pH. This is likely due to favorable electrostatic interactions between the negatively charged adsorbent and the neutral molecular form of *p*NP under mildly acidic conditions. This behavior could be due to the pK_a_ value of *p*NP (7.15), indicating that below this pH it predominantly exists in its neutral molecular form^40^. When pH ˂ pK_a_, the molecular *p*NP favors adsorption due to reduced electrostatic repulsion. At alkaline range (pH 8–12), the performance declines sharply. Since the pH_PZC_ of PSAC is 4.03 (Fig. S3), the surface remains negatively charged at pH values above this point. At pH > pK_a_, *p*NP progressively deprotonates to form the *p*-nitrophenolate anion, increasing the fraction of negatively charged species in solution^41^. The negatively charged PSAC then repels these anions electrostatically, and abundant OH^−^ competes for adsorption sites, further suppressing adsorption. Under alkaline conditions, both the PSAC surface and phenolate ions carry negative charges, resulting in strong electrostatic repulsion between them. These combined effects lowered both %R and q_e_ at high pH values, highlighting the advantage of operating near pH 6 to maximize favorable interactions. A similar trend was reported for nitrogen-doped reduced graphene oxide, which showed the highest *p*NP removal efficiency at a pH of 4–6^42^.

#### Effect of dosage

As the dosage was increased, the removal efficiency improved significantly, reaching 0.50 g L^− 1,^ as depicted in Fig. 4b. At 0.20 g L^− 1^, the removal reached 69.78% with a q_e_ of 87.22 mg g^− 1^. At 0.1 g L^− 1^ dosage, q_e_ is high at 99.56 mg g^− 1^, but the removal efficiency remains low. However, increasing the dosage to 0.5 g L^− 1^ lowered q_e_ to 42.59 mg g^− 1^ despite the high removal efficiency. This inverse relationship between q_e_ and dosage may be due to the clustering of active sites, which reduces the available surface area for adsorption per unit mass of adsorbent. Based on these observations, a dosage of 0.20 g L^− 1^ represents a practical balance between q_e_ and the removal efficiency. A similar dose-dependent pattern has been reported in *p*NP adsorption studies using leaf-based biochar^43^ and pine sawdust biochar^44^.

#### *p*NP concentration effect

At 10 mg L^− 1^ *p*NP concentration (Fig. 4c), the removal was higher, and equilibrium was reached sooner because plenty of active sites were available for the fewer *p*NP molecules. When the concentration was raised to 50 mg L^− 1^, the adsorption performance dropped, as the sites on the PSAC surface became occupied more quickly, resulting in saturation of adsorption sites and leaving excess *p*NP in solution. Similar trends have been reported for activated carbon derived from date pits in *p*NP adsorption^45^ and for powdered *Opuntia ficus-indica* used for 2-nitrophenol removal^46^. Among the tested concentrations, 25 mg L^− 1^ offered a good balance, giving reasonable removal while still allowing enough interaction without saturating the surface early.

### Adsorption modelling studies

#### Isotherm modelling

The Langmuir isotherm curve shown in Fig. 4d closely matches the experimental data, indicating a strong agreement between the model and the observed adsorption behavior. Table 1 shows that the Langmuir model achieved the highest R^2^ value (0.9884) and the lowest ꭓ^2^ (9.306), confirming its superior fitting performance. The Freundlich model also exhibited a comparatively high R^2^ (0.9876) with a slightly higher ꭓ^2^ (10.002), suggesting a minor deviation from the experimental trend, likely reflecting a degree of surface heterogeneity. The Temkin model provided a comparable fit (R^2^ = 0.9856), but its higher ꭓ^2^ value (11.553) indicates poor predictive accuracy compared to the other models. The analysis confirms that *p*NP adsorption onto PSAC proceeds predominantly via monolayer formation on a homogeneous surface, consistent with the Langmuir mechanism under the studied conditions^47^.

**Table 1:** Adsorption behaviour of PSAC toward *p*NP: isotherm and kinetic parameters.

Adsorption isotherm		
Models	Parameters	Values
Langmuir	q_m_ (mg g^− 1^)	142.933
	K_L_ (L mg^− 1^)	0.100
	χ^2^	9.306
	R^2^	0.9884
Freundlich	K_F_ ((mg g^− 1^)/(L mg^− 1^)^1/n^)	18.217
	1/n	0.551
	χ^2^	10.002
	R^2^	0.9876
Temkin	B_T_ (J mol^− 1^)	32.280
	K_t_ (L mg^− 1^)	0.918
	χ^2^	11.553
	R^2^	0.9856

Adsorption kinetics		
Models	Parameters	Values
Pseudo First Order	k_1_ (min^− 1^)	0.025
	q_e_, _Cal_ (mg g^− 1^)	34.627
	χ^2^	0.905
	R^2^	0.9974
Pseudo-Second Order	K_2_ (g mg^− 1^ min^− 1^)	7.134 × 10^− 4^
	q_e_, _Cal_ (mg g^− 1^)	40.855
	χ²	0.477
	R^2^	0.9986
Intraparticle diffusion	K_diff_ ((mg g^− 1^) min^0.5^)	2.244
	χ²	23.225
	R^2^	0.9343
	C (mg g^−−1^)	4.5178

Moreover, the separation factor (R_L_), calculated using Eq. (14), was found to be 0.286 for 25 mg L^− 1^ of *p*NP solution.

14 $${R}_{L}=\frac{1}{1+{C}_{i}{K}_{L}}$$

As the value falls within the range of 0–1, it confirms that adsorption is favorable^48^. The q_m_ determined from the Langmuir isotherm model was 142.93 mg g^− 1^, which is higher than the values reported in many previous studies employing AC for *p*NP removal (Table 2**).** This superior performance might be due to the combined effect of high porosity and the introduction of favorable surface functionalities through H_3_PO_4_ activation, underscoring the crucial role of precursor selection and activation strategy in designing efficient adsorbents under relatively mild synthesis conditions. This demonstrates the importance of precursor selection and optimized synthesis conditions, as similar activating agents can lead to very different outcomes depending on the source material.

**Table 2:** ACs synthesised from various biomass sources under different preparation conditions for the removal of *p*NP.

Sl. No.	Source of adsorbent	Synthesis conditions	SSA (m^2^ g^− 1^)	Adsorption conditions				q_m_ (mg g^− 1^)	References
				pH	Dosage (g L^− 1^)	Concentration (mg L^− 1^)	Temperature (°C)		
1	Olive cake	KOH, 600 °C, 1 h	672	7	8	5–15	25	1.55	^65^
2	Alhagi	KOH, 600 °C, N_2_, CO_2_, 2 h	641.60	8	-	30	20	27.45	^66^
3	Cherry and date stones	H_3_PO_4_, Microwave irradiation, 900 °C, 75 min	450.4 &350	-	6	100–400	-	43.5 & 45.1	^21^
4	Ravenna Grass	KOH, 700 °C, 1 h	919	7	0.5	400	25	50.89	^67^
5	Waste Orange Peels	H_3_PO_4_, 350 °C, 1 h	540.61	6	5	50	30	73.35	^27^
6	Date pits	H_3_PO_4_, N_2_, 500 °C, 1 h	-	-	2.4	115	20	108.7	^68^
7	*P. vera* shells	H_3_PO_4_, 400 °C, 2 h	670.25	6	0.2	25	30	142.93	This study

#### Kinetic modelling

Based on the provided model parameters given in Table 1, the PSO model yields the best fit, with the highest R^2^ value (0.9986) and the lowest χ^2^ (0.477). The PSO curve shown in Fig. 4e accurately follows the experimental points, particularly in the later stages. The PSO rate expression assumes that the adsorption rate is proportional to the square of the number of unoccupied sites, indicating that site availability governs the kinetics of adsorption^49^. The PFO model also showed a good fit (R^2^ = 0.9974, χ^2^ = 0.905), but slightly undervalued adsorption at intermediate times. The IPD model (R^2^ = 0.9343, χ^2^ = 23.225) poorly described the entire adsorption process, significantly deviating from the experimental data in the early and middle time ranges. This suggests that while intraparticle diffusion may occur, it is not the sole rate-controlling mechanism. The kinetic data support the PSO model as the appropriate representation of *p*NP adsorption onto PSAC, suggesting that the adsorption process is predominantly governed by surface-controlled interactions and site availability. This is consistent with the findings reported for MgCl_2_-impregnated activated carbon used for *p*NP removal^50^.

#### Thermodynamic studies

The variation of the equilibrium constant (K_T_) with temperature is illustrated in Fig. 4f and was analysed using the Van’t Hoff equation to evaluate the thermodynamic parameters. With increasing temperature, K_T_ decreases, confirming temperature dependence and indicating that lower temperatures favour stronger adsorption affinity. The ΔG° value was negative at all studied temperatures, with values of − 5.5302, − 5.2554, − 4.7133, and − 4.8236 kJ mol^− 1^ at 293, 303, 313, and 323 K, respectively, confirming spontaneity^51^. From the Van’t Hoff plot, ΔH° was calculated as − 13.428 kJ mol^− 1^, indicating an exothermic process^52^. The relatively low value of ΔH° further suggests that the interaction is predominantly physisorption^53^, while the kinetic model indicates that the process is surface-controlled. The negative standard entropy change (ΔS° = −27.102 J mol^−1^K) suggests decreased randomness at the solid-liquid interface because of the ordered arrangement of *p*NP on PSAC^54^. The model exhibited good statistical reliability, as reflected by a low χ^2^ value of 0.2296 and a high R^2^ of 0.9498, indicating a good fit between experimental and predicted values.

### Regeneration results

**Fig. 5:**
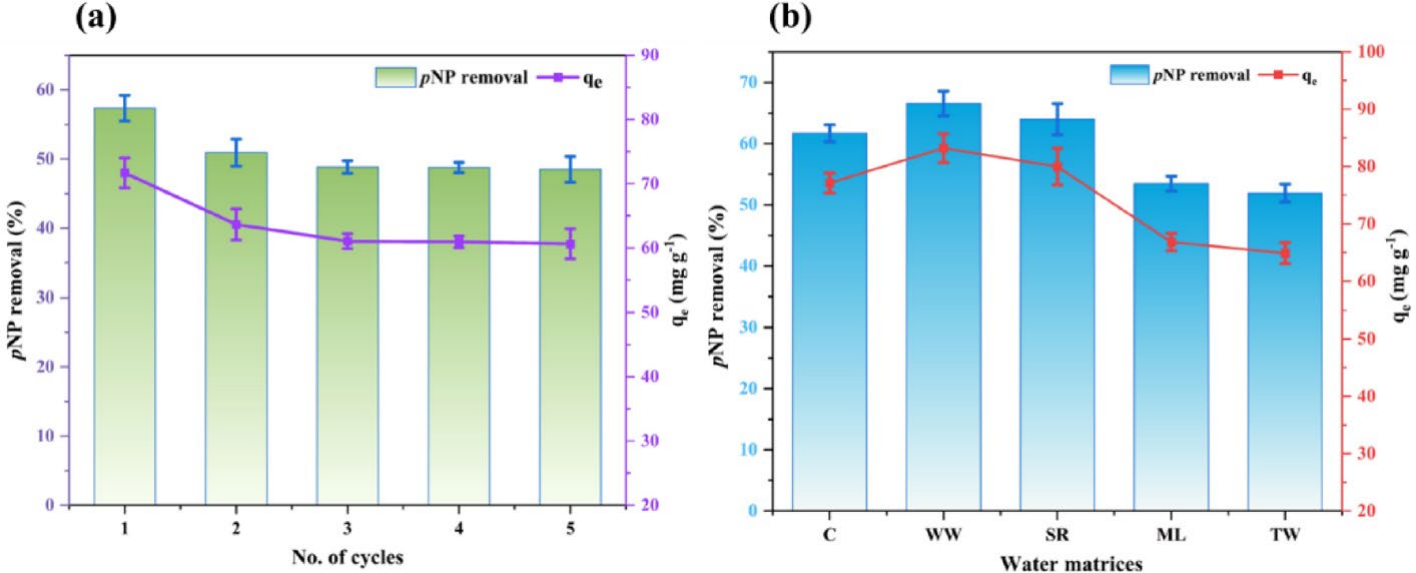
**(a)** Reusability study of PSAC for *p*NP removal, and **(b)** Effect of various water matrices (C: Control, WW: Well Water, SR: Suvarna River Water, ML: Manipal Lake Water, TW: Tap Water) on the adsorption performance of PSAC.

Figure 5a depicts the regeneration behaviour of PSAC over five adsorption-desorption cycles using 0.1 N NaOH. In the first cycle, PSAC achieved its highest performance, with 57.35% *p*NP removal and a qₑ of 71.69 mg g^− 1^. A decline appeared in the second cycle, where q_e_ decreased to 63.65 mg g^− 1^ and removal dropped to 50.92%. This decline indicates that the most significant loss in adsorption performance occurred after the first regeneration. From the third to the fifth cycles, the q_e_ remained relatively stable, ranging between 61.05 and 60.62 mg g^− 1^, and *p*NP removal percentages stabilised at 50.92 and 48.49%. The decline in performance may result from partial recovery of active sites during desorption, gradual structural degradation of the adsorbent, or diminished binding affinity after repeated reuse^55^. Compared to the semi-coke-derived adsorbent, which retained 79.5% of its initial capacity after four cycles^56^. The PSAC demonstrated superior regenerability by maintaining 85% of its original adsorption capacity even after five cycles, highlighting its strong potential for repeated use in *p*NP removal.

### Spiking studies

Figure 5b presents the adsorption performance of PSAC for removing *p*NP from various water matrices. In the control (C), *p*NP removal reached 61.7%, with the highest q_e_ of 77.12 mg g^− 1^. The WW and SR samples exhibited high removal efficiencies of 66.57% and 63.98%, respectively, and their adsorption capacities were 83.21 mg g^− 1^ and 79.98 mg g^− 1^ respectively, which were higher than that of the control. The ML and TW samples showed moderate removal efficiencies of 53.46% and 51.91%, with adsorption capacities of 66.82 mg g^− 1^ and 64.88 mg g^− 1^, respectively. Such differences among complex matrices likely stem from variations in the dissolved organic matter (DOM) and ionic composition. DOM can hinder adsorption on PSAC by competing for active sites and blocking pores^57^. In some cases, a higher ionic strength may promote phenolic adsorption by compressing the electrical double layer and reducing repulsion^58^. The greater adsorption observed in WW and SR compared with C may reflect weaker DOM interference or more favorable ionic conditions, while ML and TW likely introduce stronger DOM competition or ionic effects that limit adsorption.

### Adsorption mechanism of *p*NP onto PSAC

The plausible adsorption mechanism of *p*NP onto PSAC is illustrated in Fig. S4, where a synergistic combination of physical and chemical interactions occurs. The superior surface area and porous architecture of PSAC help *p*NP molecules move across the boundary layer and further diffuse into its internal pores, enabling efficient mass transfer^59^. Specific chemical interactions like hydrogen bond interactions among surface -OH/‐CO groups of PSAC and the ‐OH/‐NO_2_ groups of *p*NP, as confirmed by FT‐IR spectral shifts. The fit of the Langmuir isotherm model and PSO kinetics further supports monolayer coverage and chemisorption-driven adsorption. At pH 6 the PSAC surface is negatively charged, while *p*NP exists predominantly in its molecular form. At pH values below 4, the PSAC surface becomes positively charged, whereas *p*NP remains neutral; thus, electrostatic attraction is minimal, and adsorption depends primarily on non-electrostatic forces. At alkaline pH, *p*NP converts to *p*-nitrophenolate ion, and because the PSAC surface is negatively charged, electrostatic repulsion occurs, leading to reduced adsorption efficiency. Thus, factors like hydrogen bonding, hydrophobic interactions, and van der Waals forces can influence the adsorption process^52^. In parallel, physisorption mechanisms, such as π‐π stacking between the aromatic domains of PSAC and *p*NP, contribute to adsorption, as evidenced by the FT-IR results^60^. The adsorption is thermodynamically favorable and exothermic in nature, monolayer adsorption, involving both physical and chemical mechanisms, as confirmed by kinetic, isotherm, and thermodynamic analyses.

### Machine learning modelling

#### ANN modelling

**Fig. 6:**
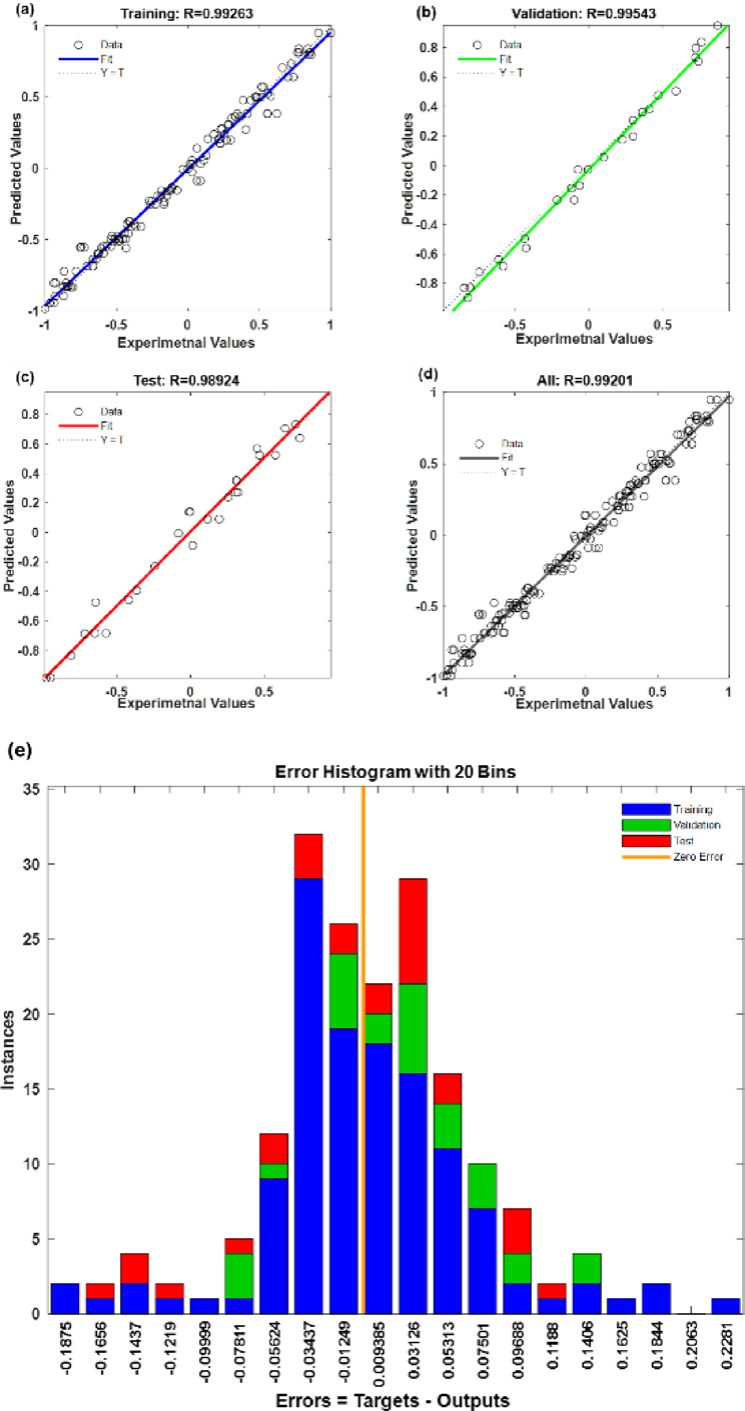
ANN Regression plot of **(a)** training, **(b)** validation, **(c)** testing, **(d)** overall, and **(e)** histogram plot for *p*NP adsorption onto PSAC.

The most suitable ANN architecture was identified as 5–10–1, comprising five input neurons, ten hidden neurons, and one output neuron representing the q_e_. To further assess the predictive accuracy of the optimised ANN structure, regression plots (Fig. 6a and d) were generated to illustrate the agreement between experimental and predicted values. The correlation coefficient (R) value for the training, validation, testing, and overall datasets were 0.99263, 0.99543, 0.98924, and 0.99201, respectively. The corresponding MSE values were 0.0044 for training, 0.0040 for validation, and 0.0060 for testing, further confirming the strong predictive ability of the network. These consistently high R values, low MSEs, with only minor variation across datasets, highlight the robustness and reliability of the model^61^. The proximity of the regression lines to the ideal fit (Y = T) demonstrates that the ANN model effectively captured the nonlinear relationships among the process variables, providing accurate forecasts of *p*NP adsorption performance. The error histogram (Fig. 6e) revealed that most residuals were clustered close to zero, with no major outliers, supporting the reliability of the ANN model^62^.

#### ANFIS model

**Fig. 7:**
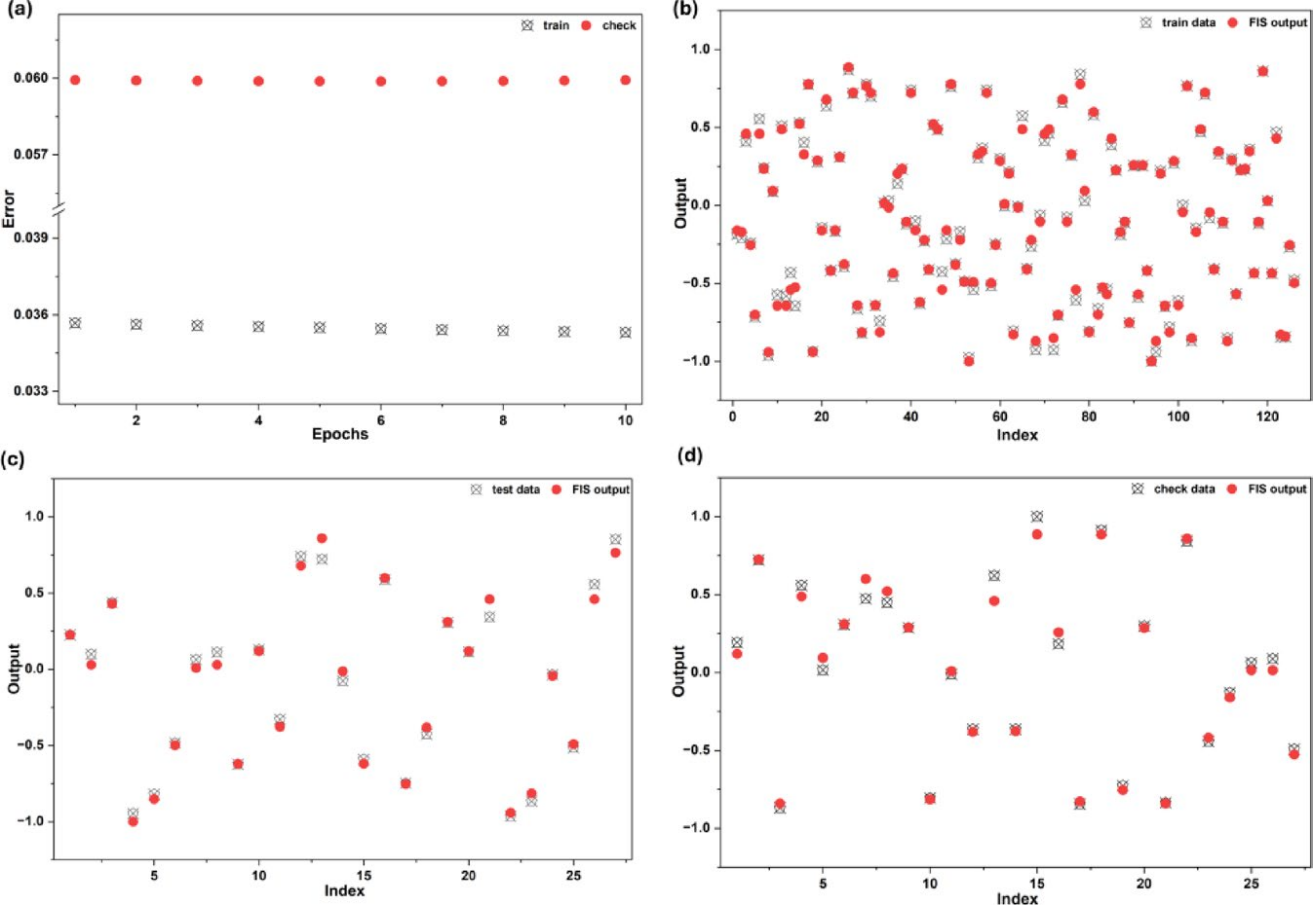
**(a)** ANFIS Training curve, Comparison of experimental and predicted values of **(b)** training, **(c)** testing, and **(d)** checking for *p*NP adsorption onto PSAC.

For each input variable, a three-membership-function configuration was used, allowing the model to capture the nonlinear behaviour of the system effectively. The output using the triangular membership function (TriMFs) is depicted in Fig. 7. As seen in Fig. 7a, the training error (0.0353) and checking error (0.0599) remained steady from the beginning and did not fluctuate much, which shows that the model was not overfitting. For the testing data, an error of 0.0562 was obtained, suggesting that the model handled both learning and generalisation reasonably well. In Fig. 7b, the scatter plot reveals that the predicted values follow the experimental results quite closely. The testing phase shown in Fig. 7c also aligns well, with predictions matching the observed data. Figure 7d presents the checking stage, where the predicted outputs align closely with the corresponding experimental data points. The close match demonstrates strong consistency between the predicted values by ANFIS and the experimental data^63^, confirming that the model is reliable for predicting the adsorption mechanism.

The ANFIS sensitivity analysis showed how each operating variable affected *p*NP adsorption by PSAC. As shown in Fig. S5a, pH followed a bell-shaped pattern, with the highest q_e_ near neutral conditions, indicating its significant effect. Adsorbent dosage (Fig. S5b) showed an opposite trend, where a higher dosage reduced the uptake per unit mass, likely because not all adsorption sites were fully utilized. Initial concentration (Fig. S5c) had a strong positive effect, as higher pollutant levels increased the driving force for mass transfer. Contact time emerged as another key factor, showing a sharp rise in capacity, followed by stabilization as equilibrium was approached (Fig. S5d). Temperature presented a curved profile, indicating improved uptake at moderate levels but possible desorption at elevated temperatures (Fig. S5e). The sensitivity ranking in Fig. S5f shows that contact time is the most influential factor. This agrees with the results from a membrane adsorption system for the adsorption of malachite green dye, where the contact time was found to be the key variable^63^. This was followed by the influence of concentration, dosage, pH, and finally temperature.

#### Evaluation and comparison of ANFIS and ANN models

**Fig. 8:**
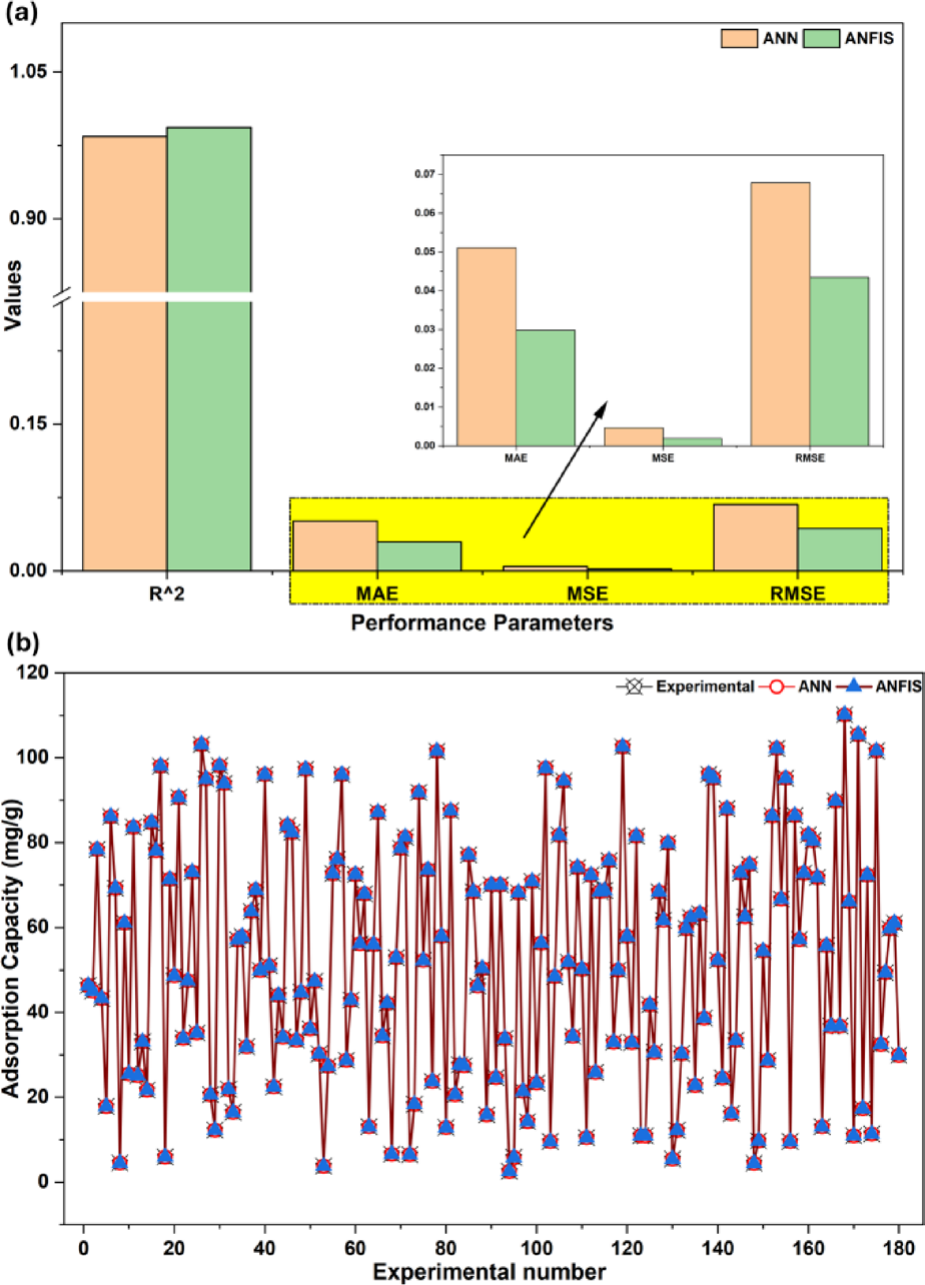
**(a)** Performance parameters of ANN and ANFIS models, **(b)** comparison of experimental and predicted values of ANN and ANFIS models.

A comparison of the ANN and ANFIS models to predict qₑ of *p*NP on PSAC showed clear differences in their performances. The performance curves in Fig. 8a show that the ANFIS predictions match the experimental data better than those of the ANN, confirming its higher reliability under different adsorption conditions. Similar trends have been noted in other systems, such as the simultaneous removal of dye and Cu(II) using sawdust, where ANFIS offered closer agreement with experimental data than ANN^64^. The plot in Fig. 8b demonstrates a good match between the predicted and measured values for both models. The statistical results in Table S1 further show that ANFIS achieved a higher R^2^ (0.9935) than ANN (R^2^ = 0.9841), underscoring its better ability to capture the adsorption mechanism. The ANFIS model reported lower error values, with MAE, MSE, and RMSE of 0.0298, 0.0019, and 0.0434, respectively, and the corresponding values for ANN were 0.0511, 0.0046, and 0.0679. It indicates that ANFIS provides a more accurate and robust description of *p*NP adsorption on PSAC.

## Conclusion

AC prepared from waste *P. vera* shells using H_3_PO_4_ proved highly effective for removing *p*NP, with a q_m_ of 142.93 mg g^− 1^. The adsorption behavior was best represented by the pseudo-second-order kinetic model, while thermodynamic parameters confirmed an exothermic and spontaneous physisorption process. Combined with the Langmuir isotherm results, these findings establish that *p*NP uptake on PSAC occurs via monolayer adsorption involving both chemical and physical interactions. The PSAC also exhibited good regeneration ability, maintaining stable performance over five adsorption-desorption cycles, and showed consistent removal efficiency across different water matrices. ML modelling with ANN and ANFIS confirmed the reliability of the experimental data, with ANFIS showing a higher predictive accuracy and lower errors. This study demonstrates that PSAC is a sustainable, economical, and effective adsorbent for *p*NP-contaminated wastewater, contributing to waste valorization while ensuring reliable pollutant removal.

## Supplementary Information

Below is the link to the electronic supplementary material.

Supplementary Material 1

## Data Availability

Data will be made available on reasonable request.
