# Supplementary material for: Machine learning and mechanistic studies on p-nitrophenol remediation using sustainable activated carbon
Source: Sci Rep. 2026 Mar 4;16:12153. doi: 10.1038/s41598-026-42718-2 (PMC13077033; doi:10.1038/s41598-026-42718-2)
Supplement: Supplementary file 1 — Supplementary Material 1 [file 41598_2026_42718_MOESM1_ESM.docx]

**List of Figures**

**Figure S1.** Overview of the preparation process of *Pistacia vera* shell-derived AC (PSAC)

**Figure S2. (a)** FT-IR spectra of PSAC before and after adsorption of *p*NP **(b)** XRD spectra of PSAC before and after the adsorption of *p*NP

**Figure S3.** Point of zero charge (pH_PZC_) of PSAC

**Figure S4.** Schematic illustration of the anticipated adsorption behaviour of *p*NP onto PSAC

**Figure S5.** Sensitivity Analysis of ANFIS model: **(a)** Effect of pH, **(b)** Effect of Dosage, **(c)** Effect of Concentration, **(d)** Effect of Time, **(e)** Effect of Temperature and **(f)** Ranking plot for *p*NP adsorption onto PSAC


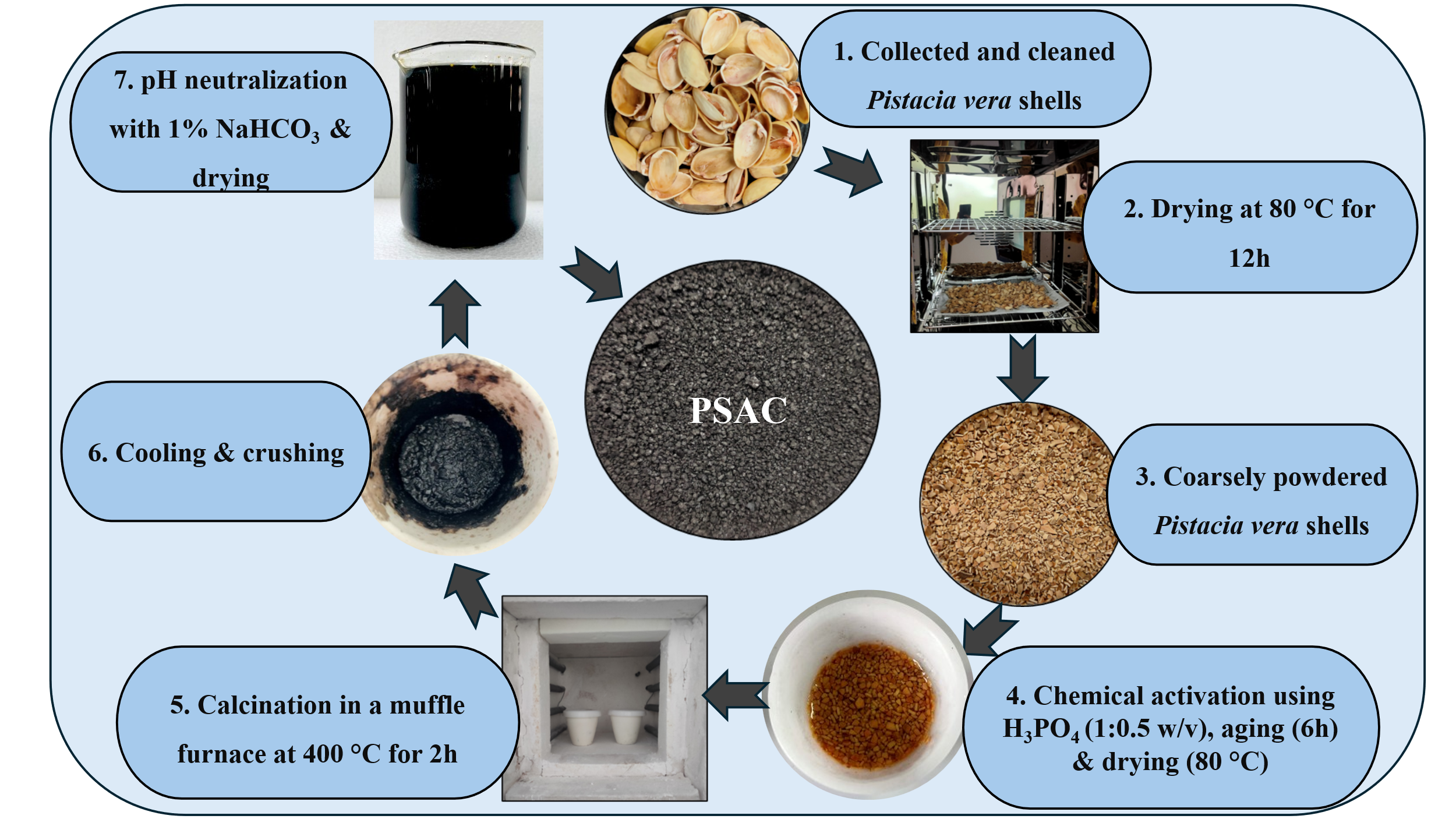


**Figure S1.** Overview of the preparation process of *Pistacia vera* shell-derived AC (PSAC)


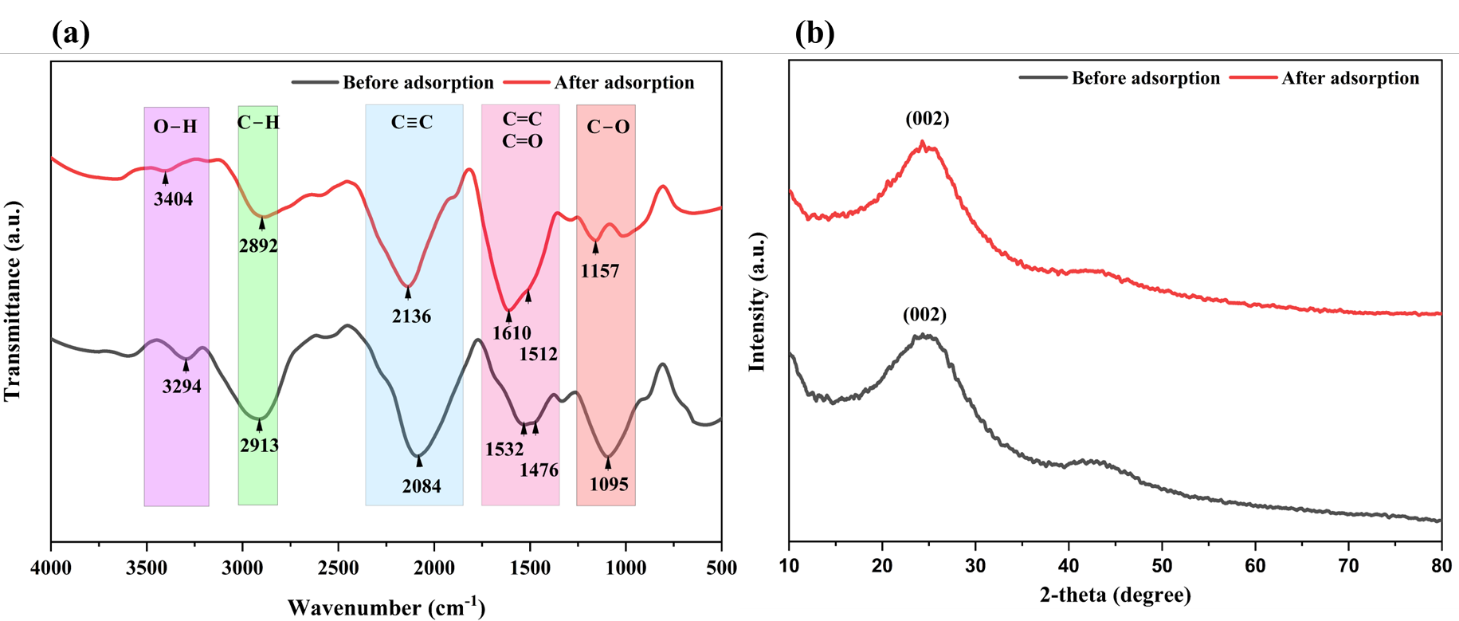


**Figure S2. (a)** FT-IR spectra of PSAC before and after adsorption of *p*NP **(b)** XRD spectra of PSAC before and after the adsorption of *p*NP

**Figure S3.** Point of zero charge (pH_PZC_) of PSAC**
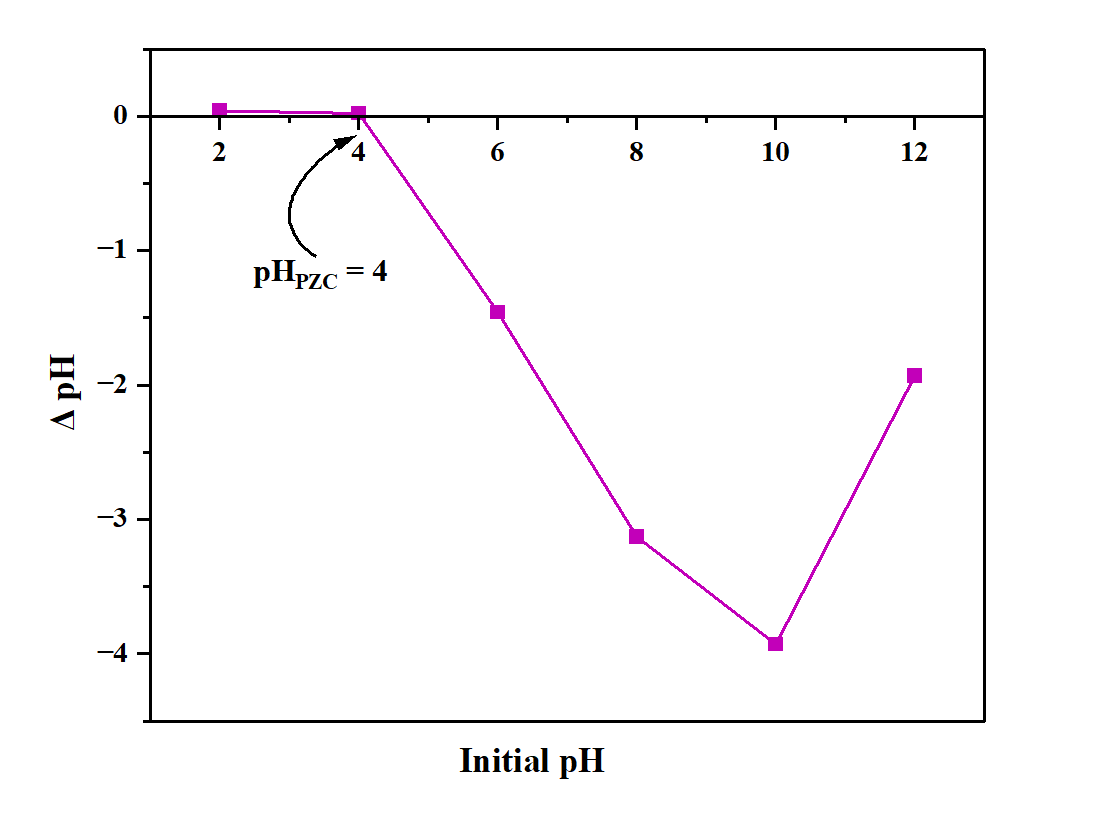
**


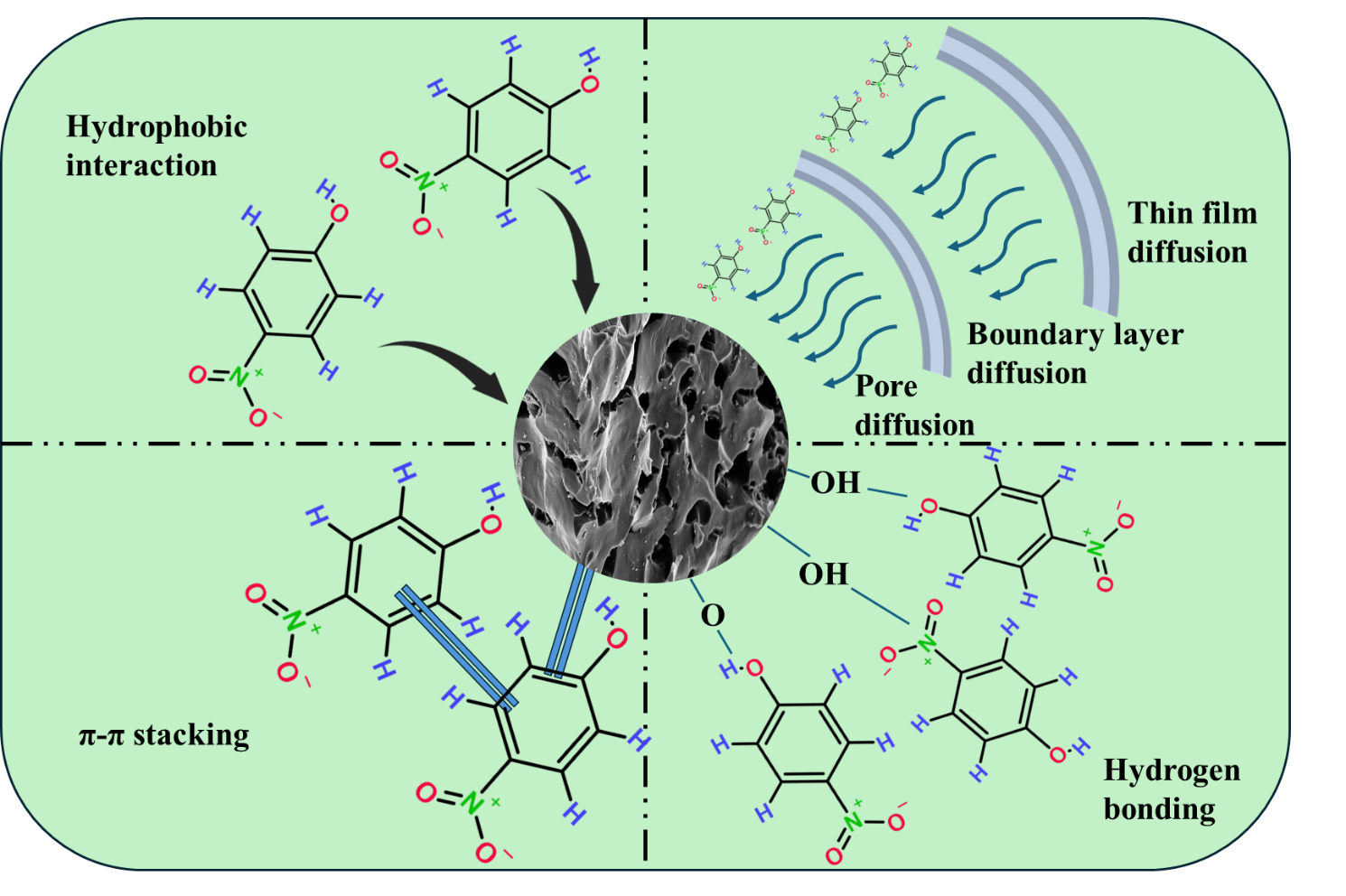


**Figure S4.** Schematic illustration of the anticipated adsorption behaviour of *p*NP onto PSAC


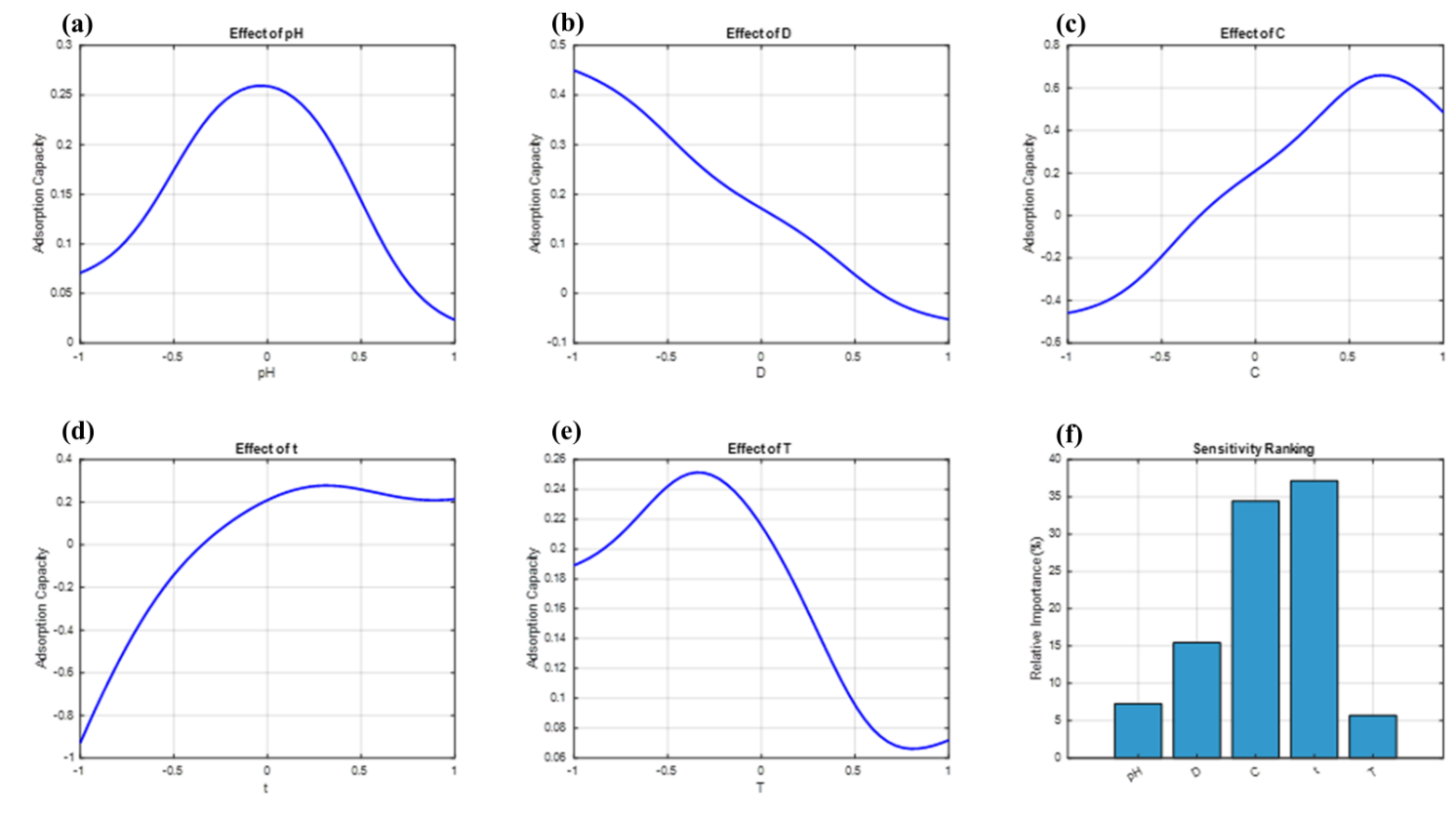


**Figure S5.** Sensitivity Analysis of ANFIS model: **(a)** Effect of pH, **(b)** Effect of Dosage, **(c)** Effect of Concentration, **(d)** Effect of Time, **(e)** Effect of Temperature and **(f)** Ranking plot for *p*NP adsorption onto PSAC

**List of Tables**

**Table S1.** Performance comparison of ANN and ANFIS models for *p*NP adsorption using PSAC

**Table S1:** Performance comparison of ANN and ANFIS models for *p*NP adsorption using PSAC

| Error evaluation metrics | ANN | ANFIS |
| --- | --- | --- |
| R^2^ | 0.9841 | 0.9935 |
| MAE | 0.0511 | 0.0298 |
| MSE | 0.0046 | 0.0019 |
| RMSE | 0.0679 | 0.0434 |
